# Efficient Exothermic Press toward Ultrafast and Scalable Manufacturing of Complex Polymer Composites

**DOI:** 10.1002/advs.202509336

**Published:** 2025-07-17

**Authors:** Amirreza Tarafdar, Haining Zhang, Xinlu Wang, Andrea J. Hoe, Kaiyue Deng, Kelvin Fu, Quinn Qiao, Ian D. Hosein, Yeqing Wang

**Affiliations:** ^1^ Department of Mechanical and Aerospace Engineering Syracuse University Syracuse NY 13244 USA; ^2^ Department of Biomedical and Chemical Engineering Syracuse University Syracuse NY 13244 USA; ^3^ Department of Mechanical Engineering University of Delaware Newark DE 19716 USA

**Keywords:** additive manufacturing, complex composite structures, continuous fiber composites, frontal polymerization, hybrid frontal and commercial epoxy resin, thermosetting resin

## Abstract

Rapid and scalable production of high‐performance composites remains a key challenge in achieving sustainable manufacturing. Here, Exo‐press frontal polymerization (EPFP), a novel and transformative method for manufacturing fiber‐reinforced thermoset polymer composites, overcoming energy efficiency, scalability, and curing complex geometries, is introduced. Unlike conventional curing methods that require prolonged processing times and high energy, EPFP utilizes exothermic heat to reduce curing time from hours to minutes with minimal external energy. Combining exothermic heat with press molding, the novel EPFP enables the efficient fabrication of complex geometries, such as airfoil skin sections, with high fiber volume fractions (above 60%). In addition, EPFP is compatible with commercial off‐the‐shelf epoxy by integrating frontal resin, showcasing its versatility and adaptability for diverse industrial applications. Composites manufactured using EPFP exhibit superior thermomechanical properties while significantly reducing energy consumption by 80% and production costs by 40%. This makes it a sustainable and efficient solution for polymer composites manufacturing.

## Graphical abstract

Exo‐press frontal polymerization is a rapid, low‐energy process to produce multiscale fiber‐reinforced polymer composites at high fiber volume fractions. By leveraging localized heat, along with a hybrid monomer system, the reaction front cures parts within a minute and forms a copolymer network. This scalable method accommodates complex geometries, such as airfoil skin section, while significantly reducing energy and costs.

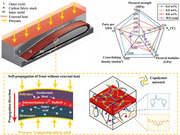

## 1 Introduction

Rapid and efficient production of high‐performance thermoset‐based fiber‐reinforced polymer composites (FRPCs), known for their excellent strength and thermal stability, is fast becoming a key area of research, as it quenches the desire from different industries to counter high costs and environmental loads implicated with conventional methods of manufacture.^[^ ^1^, ^2^, ^3^, ^4^ ^]^ However, conventional FRPCs manufacturing relying on prolonged energy‐intensive thermal cycles and complicated fabrication process are suffering from high cost and withdraws large‐scale manufacturing.^[^ ^5^ ^]^ For instance, curing a section of an autoclaved carbon fiber/epoxy fuselage for Boeing 787 requires about 350 gigajoules (GJ) of energy for an 8‐h cycle, perchance emitting more than 80 tons of carbon dioxide.^[^ ^6^ ^]^ Beyond the environmental and economic burdens, these methods also demand complex experimental setups to fabricate complicated geometries, further constraining design flexibility and slowing innovation. For example, the 777 facility required a $1 billion investment in autoclaves measuring 37 m long and 9 m in diameter, constraining maximum part size and curvature to the vessel envelope.^[^ ^7^ ^]^ Wing skins, for instance, must be segmented to fit within autoclaves, limiting the manufacturing of large continuous aerodynamic surfaces.^[^ ^7^, ^8^ ^]^ These energy‐intensive processes make scaling of manufacturing prohibitively high, thus calling for innovations toward transformative fast, energy‐efficient, and sustainable alternatives.

Frontal polymerization (FP) has revolutionized the process of composite manufacturing. The self‐propagating reaction front uses little more than a localized heat trigger to initiate the process, and due to the exothermic nature of polymerization, it continues the propagation of a reaction front.^[^ ^9^, ^10^ ^]^ FP has already been used successfully in the fabrication of a variety of polymeric materials including functionally graded polymers, nanocomposites, and FRPCs.^[^ ^11^, ^12^, ^13^, ^14^, ^15^, ^16^, ^17^ ^]^ However, difficulties arise when applying FP for high‐performance epoxy‐based FRPCs. The high fiber volume fractions can inhibit front propagation because of the network of densely packed fibers acting as a thermal sink, hence upsetting the delicate balance between exothermic heat generated during FP and heat loss to the surroundings to propagate the reaction.^[^ ^18^ ^]^ While acrylate monomers are more reactive and suitable for FP, they have a short pot‐life for a few hours, which limit their application in structural needs.^[^ ^9^ ^]^ Epoxy monomers on the other hand led to the formation of mechanically strong composites with longer pot‐life but are less reactive, hence forming the basis of great difficulty in FP.^[^ ^19^, ^20^, ^21^ ^]^ Thus, a new approach is needed to address epoxy's inherently lower reactivity, particularly in high fiber volume domains while retaining epoxy's superior mechanical properties. Recent attempts to achieve high *V*_f_ have faced significant hurdles in terms of fabrication complexity, propagation control, and scalability. Tran et al.^[^ ^22^ ^]^ and Vyas et al.^[^ ^23^ ^]^ have been able to achieve *V*_f_ between 35% and 65%; however, they have been hampered by process complexity or temperature management issues. Specifically, Vyas et al.^[^ ^23^ ^]^ demonstrated through‐thickness FP of carbon fiber composites using a highly reactive dicyclopentadiene (DCPD) resin, with resistive heating and modest isostatic pressure (≈0.3 MPa) to achieve a *T*_g_ of ≈156 °C in 65% *V*_f_ laminates. However, this approach relies on a highly exothermic DCPD resin system and continuous heating, contrasting with our Exo‐press frontal polymerization (EPFP) process with an epoxy resin system. In addition, prior studies have demonstrated the potential of radical‐induced cationic FP of epoxy frontal resins and composites, including UV‐activated and dual initiator systems,^[^ ^19^, ^24^ ^]^ these efforts largely focus on neat resin systems or fiber‐reinforced composites with simplified geometries. For instance, Gachet et al.^[^ ^24^ ^]^ and Staal et al.^[^ ^18^ ^]^ improved it, achieving a *V*_f_ of around 60%, using thermal preheating techniques and sacrificial resin channels. These techniques, while effective, do not lend themselves to straightforward manufacturing nor scalable processes.

We tackle these challenges by introducing a novel EPFP technique. By providing localized thermal triggering with mechanical compaction, EPFP allows the propagation of front and avoids front quenching within a highly dense fiber domain, facilitating the efficient manufacture of FRPCs containing beyond 60% *V*_f_, including those with complex geometric shapes. EPFP method removes the need for any sacrificial resin channels, simplifies the process, and maintains viability for industrial‐scale implementation. Building on this advancement, we further extend the applicability of EPFP by hybridizing the frontal resin with off‐the‐shelf West System (WS) epoxy, a widely used commercially off‐the‐shelf epoxy system toward high *V*_f_ fiber reinforced composites. This novel hybridization allows us to leverage the rapid curing efficiency of EPFP to dramatically reduce curing and hence manufacturing times for industrial‐grade epoxy laminates while preserving the superior mechanical properties of conventional curing methods. The combination of high *V*_f_, rapid curing, and simplified fabrication process places EPFP in a realm of possibility for the revolution for much greener manufacturing of composites.

## 2 Result and Discussion

### 2.1 Overcoming Low Reactivity of Epoxy‐Based Frontal Resin in Complex Geometry via EPFP

**Figure 1:**
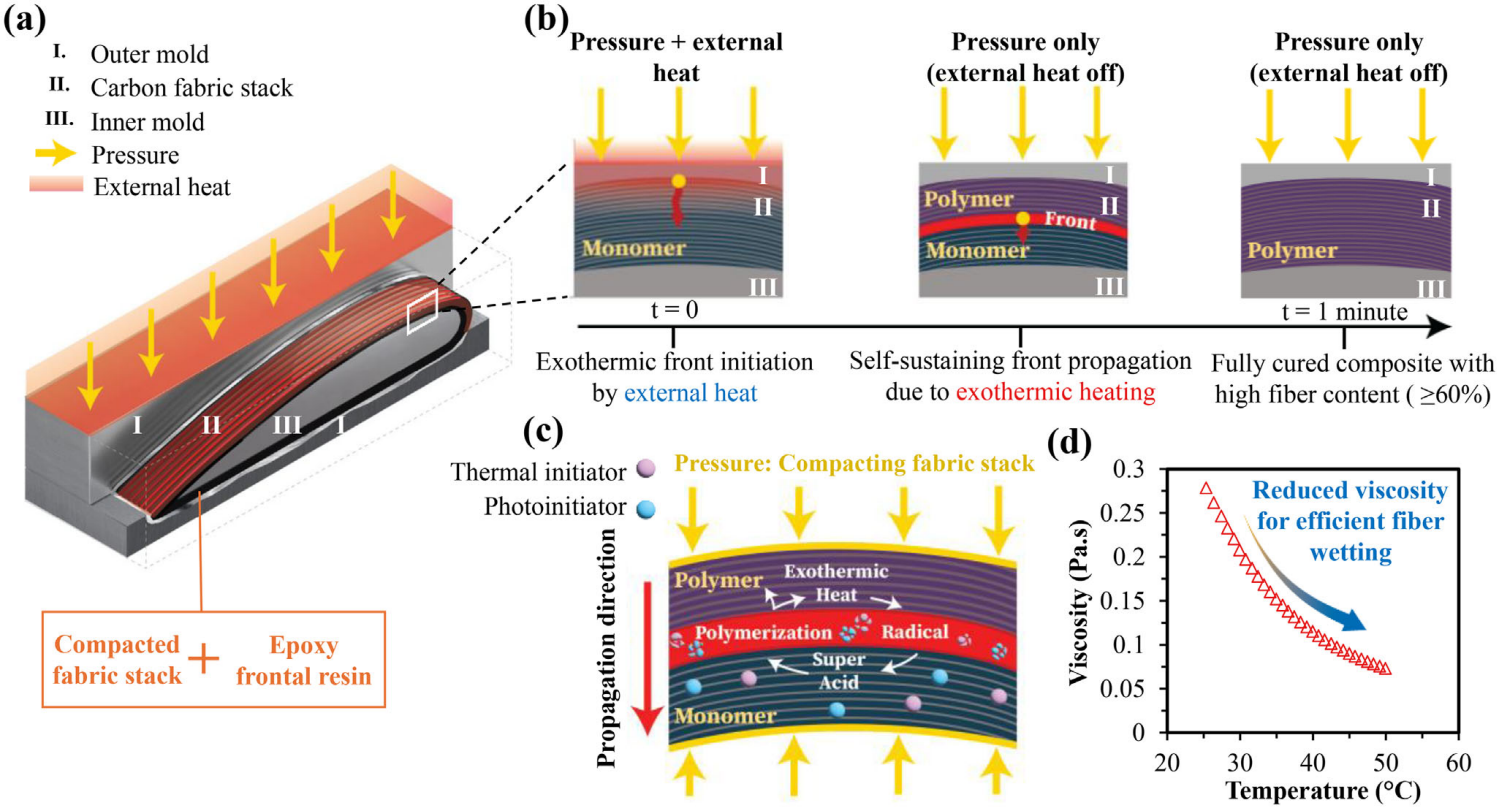
Concept of exothermic press frontal polymerization (EPFP) process. a) EPFP approach for airfoil‐shaped CFRP fabrication. A hot press applies pressure and localized heat to initiate polymerization in a resin‐impregnated carbon fiber stack, with the reaction proceeding self‐sufficiently once the exothermic front is established. b) Progression of the polymerization front over time. The exothermic reaction propagates throughout the laminate, transitioning the monomer to polymer. No external heating is required beyond the initial thermal trigger. c) Self‐sustaining reaction cycle. Thermally generated super acids and radicals drive ring‐opening polymerization, continuously releasing heat that supports front propagation in a compacted fabric stack without front quenching. d) Viscosity–temperature relationship. Preheating the frontal resin to 50 °C substantially lowers viscosity, promoting efficient fiber wetting and enabling high fiber volume fractions in the cured laminate.

Thermoset epoxy monomers are well known for their superior mechanical properties yet relatively low reactivity in FP. This poses a significant challenge in high fiber volume fraction (*V*_f_) composites, where the dense fiber network can rapidly dissipate heat, potentially causing front quenching. **Figure** **1** introduces our EPFP method as a robust approach to circumvent these limitations, enabling rapid, self‐propagating curing front in epoxy‐based composites, even in complex geometries such as an airfoil skin.

Figure 1 schematically depicts how EPFP addresses the challenges of low‐reactivity epoxy systems. In particular, Figure 1a shows the press‐based setup in greater detail, where a hot press applies pressure (i.e., 8 MPa) and localized heat to a resin‐impregnated carbon fiber stack with an airfoil geometry, initiating the polymerization front. The stack was prepared by wet hand lay‐up, with the resin pre‐warmed and degassed at 50 °C to remove bubbles, reduce viscosity, and ensure thorough impregnation before the mold was closed. Once triggered in a few seconds, the exothermic heat generated by the curing reaction sustains the front while the external heat source is switched off, drastically reducing the need for prolonged external heating. By coupling the thermal triggering with mechanical pressure, EPFP ensures excellent consolidation of the laminate and maintains the temperature threshold necessary for continuous front propagation. The temporal progression of this front is illustrated in Figure 1b, where the curing front advances through the thickness of the laminate within a minute, which is substantially faster than traditional autoclave or oven‐based epoxy curing. Noted that the curing time took a minute including the preheating and polymerization stages to make sure that the laminate is fully cured. Despite the low reactivity typical of epoxy‐based frontal systems, the EPFP approach establishes and sustains a sufficiently high reaction temperature to avoid quenching in a highly dense fiber domain.

Fundamental to achieving this self‐sustaining process is a chain of chemical reactions, illustrated in Figure 1c. Thermally activated initiators decompose to generate highly reactive species, such as super acids or radicals, which in turn catalyze ring‐opening polymerization. Specifically, the thermal triggering decomposes the iodonium salt in the photoinitiator of the frontal resin, generating a strong super acid via the photoacid generator (PAG). The generated super acid initiates ring‐opening polymerization, leading to polymerization and the release of exothermic heat. The released heat also decomposes the thermal initiator (TID), creating free radicals that further drive the PAG cycle, sustaining the polymerization process without requiring continuous external energy input. The exothermic heat from these reactions push the front to propagate through the thickness, while the preheated resin and mold mitigate heat‐loss effects that would otherwise quench epoxy‐based FP at high *V*_f_. By precisely tuning initiator concentrations and press temperature, EPFP effectively counteracts the low intrinsic reactivity of epoxy monomers, yielding uniformly cured composites. In addition, preheating the resin to 50 °C lowers its viscosity from 0.27 to 0.07 Pa s (see Figure 1d), promoting efficient fiber wetting and minimizing the likelihood of local front quenching in high *V*_f_ domains. Together, the efficient fiber wetting and controlling heat dissipation via EPFP technique ensure a stable, fully propagated front across the entire laminate thickness.

Building on this foundation, we further demonstrate EPFP's versatility by formulating a hybrid resin, mixing the epoxy frontal resin with a commercial off‐the‐shelf WS epoxy. While EPFP itself resolves the front‐quenching dilemma in dense laminates, the hybrid approach provides a flexible pathway for fine‐tuning mechanical performance and industrial adaptability of epoxy‐based FRPCs. Although Figure 1 highlights EPFP's potential for a complex airfoil‐shaped part, the mechanical and thermal characterizations presented later in this study were conducted on standardized flat‐beam laminate coupons for both neat frontal and hybrid resin compositions. This strategy offers well‐controlled test conditions while maintaining the practical relevance of high *V*_f_ epoxy systems. In a later section, we demonstrate the full fabrication of an airfoil component for underscoring EPFP's scalability and applicability to advanced aerospace manufacturing.

### 2.2 Numerical Validation of the Exo‐press Frontal Polymerization Process

**Figure 2:**
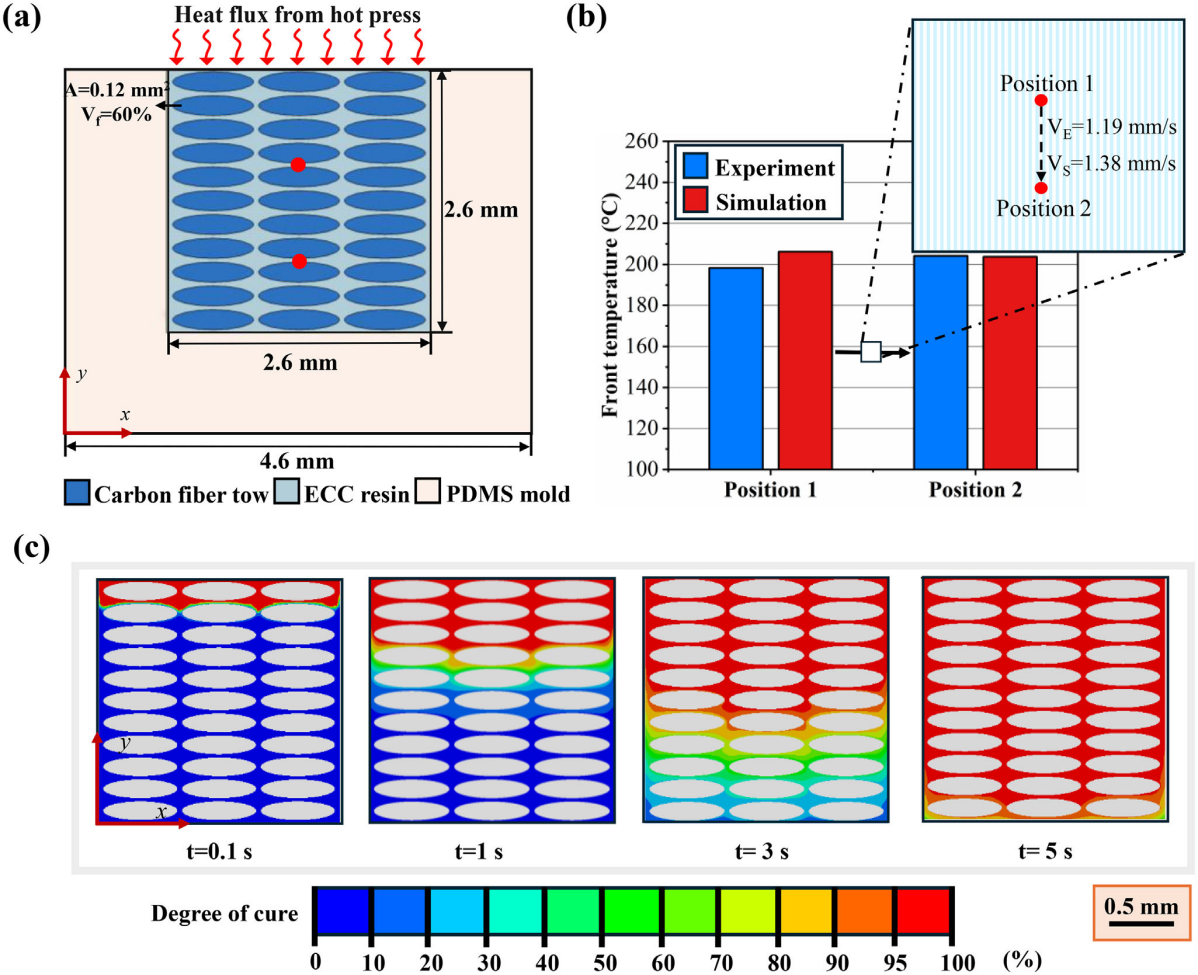
Comparison of experimental and simulation results for frontal polymerization in EPFP process. a) Schematic of the simulation setup for the EPFP process. A CFRP laminate (*V*_f_ = 60%) inside a PDMS mold, with a heat flux applied from the top surface to initiate the polymerization. b) Comparison of front temperature. Experimental and simulation (denoted as subscript E and S, respectively) results are shown for front temperature and through‐thickness front velocity at two positions in the laminate. c) Evolution of the degree of cure over time. Curing front propagates through the thickness under 200 °C triggering temperature, showcasing a self‐sustainable front propagation.

To gain insights into the influence of triggering temperature on the EPFP process, we performed a two‐dimensional (2D) numerical simulation incorporating heat transfer, curing kinetics, and internal heat generation from the exothermic polymerization reaction. **Figure** **2**a shows the simulation setup, which uses a 2.6 mm × 2.6 mm 2D geometry representing a cross‐section of the CFRP laminates. Elliptical fiber tow arrangements were implemented to achieve an overall fiber volume fraction (*V*_f_) of 60%, consistent with the experimental samples. This geometry captures key bulk properties and enables accurate modeling of heat transfer and reaction dynamics, as validated by prior studies.^[^ ^3^ ^]^ In the simulation, fiber tow regions were modeled as solid carbon fiber excluding intra‐tow resin for computational simplicity. Consequently, all heat generation was attributed to the inter‐tow resin regions. This approach, consistent with prior work validated against experimental data,^[^ ^25^ ^]^ captures the dominant thermal and reaction phenomena in the EPFP process despite this simplification. A localized heat flux is applied at the top surface to initiate polymerization. The simulation incorporates curing kinetics parameters extracted from DSC data for 0.4 wt% PI (Figure S1 and Table S1, Supporting Information) and uses the Arrhenius‐based Prout–Tompkins autocatalytic model to capture the evolution of the degree of cure over time. Noted that for validation, the laminate was cured in a PDMS inner mold encased in a rigid aluminum frame, so the simulation boundary conditions match the actual experimental setup for monitoring FP process.

To validate the acquired curing kinetics and the simulation, we compared experimental measurements and predicted values for front velocity and temperature at two distinct positions (Figure 2b). Experimentally, the though thickness front velocity measured 1.19 mm s^−1^ (±0.31 mm s^−1^), while the simulation predicted 1.38 mm s^−1^, resulting in about a 15% deviation. At position 1, the measured front temperature averaged 194.9 °C (±4.8 °C), closely matching the simulated 206.1 °C (3% deviation). Similarly, at position 2, the experimental front temperature of 196.0 °C (±9.1 °C) aligned well with the simulated 203.8 °C (3.9% deviation). These results confirm the model's ability to accurately represent the evolution of the FP process, as well as the reliability of the calculated curing kinetics.

Building on this validated model, we next explored how the triggering temperature affects cure completeness. We performed a parametric study varying the triggering temperature (150, 175, and 200 °C) to identify conditions that promote fully curing through the laminate thickness (Figures S2 and S3, Supporting Information). Under a 200 °C triggering temperature, the degree of cure rapidly approached 100% across the laminate (Figure 2c), whereas lower initiation temperatures yielded slower front propagation and partial curing (Figures S2 and S3, Supporting Information). These findings emphasize the importance of choosing an appropriate initiation temperature to ensure a stable, through‐thickness front propagation. Thus, a triggering temperature of 200 °C was selected for all subsequent experimental studies, since it guaranteed a fully consolidated laminate and complete propagation of the front through the thickness, even in thermally conductive aluminum molds. While this temperature ensures successful propagation, the final degree of cure is governed by photoinitiator content, as evidenced in both simulation and experimental results.

### 2.3 Photoinitiator Tuning for Optimization of Characteristics of EPFP Fabricated Laminate

**Figure 3:**
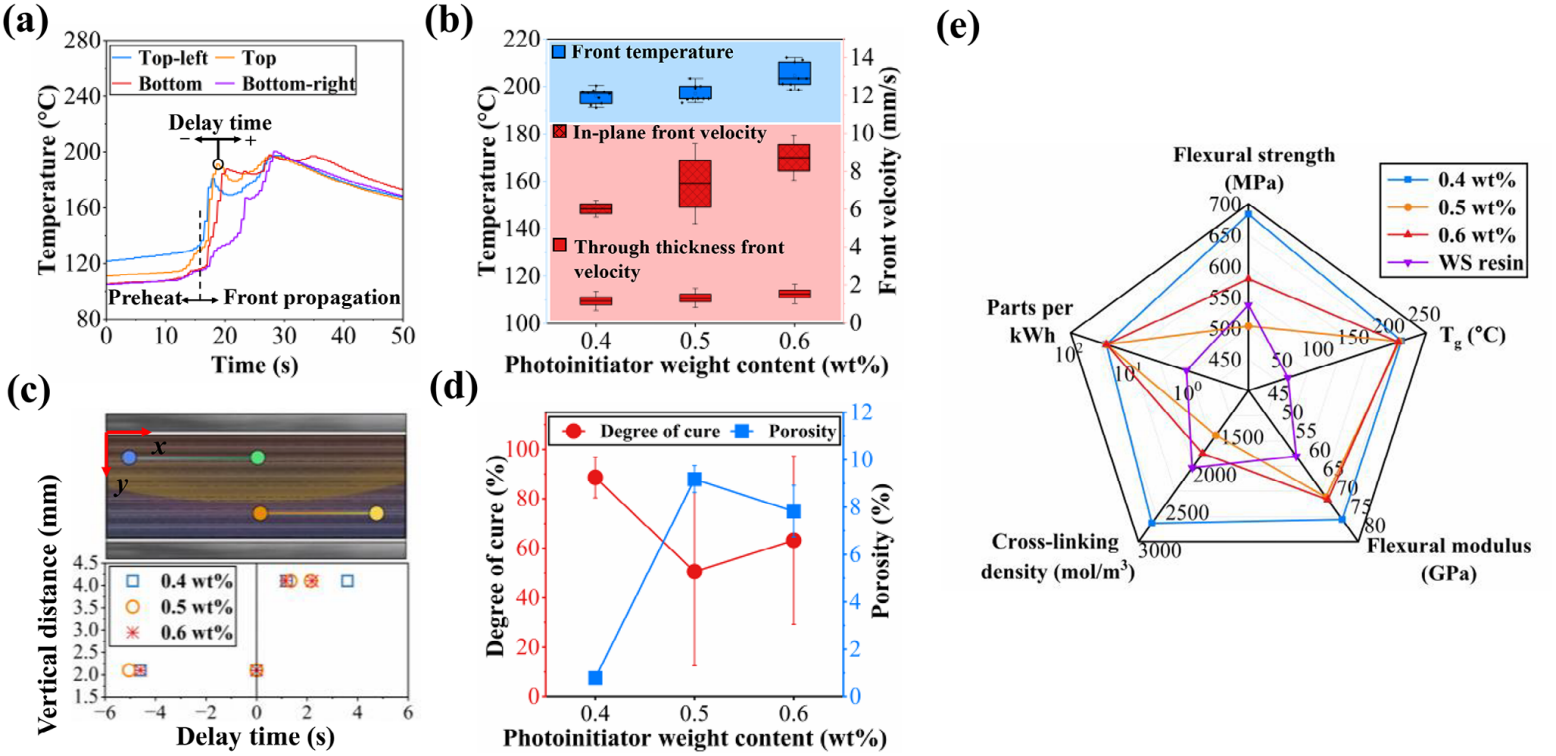
Influence of photoinitiator (PI) content on characteristics of EPFP process and thermomechanical properties of CFRP laminates with neat frontal resin. a) Temperature profile related to 0.4 wt% PI laminate. The top center thermocouple in the laminate serves as the reference, and the delay time for front arrival at other positions is defined in relative to this point. b) Front temperature and front velocity measurement in different directions. Front temperature and front velocity in through‐thickness and in‐plane direction vary as a function of PI content (0.4, 0.5, and 0.6 wt%). c) Delay time at different points through the laminate thickness. Delay time measured for the polymerization front to reach different depths within the laminate, referenced to the top center thermocouple, illustrating the impact of PI content on front propagation. d) Porosity and degree of cure for laminates fabricated with 0.4, 0.5, and 0.6 wt% PI. e) Thermomechanical properties and manufacturing rate. The chart shows the effect of PI content on thermomechanical properties and rate of manufacturing for the CFRP laminates.

**Figure 4:**
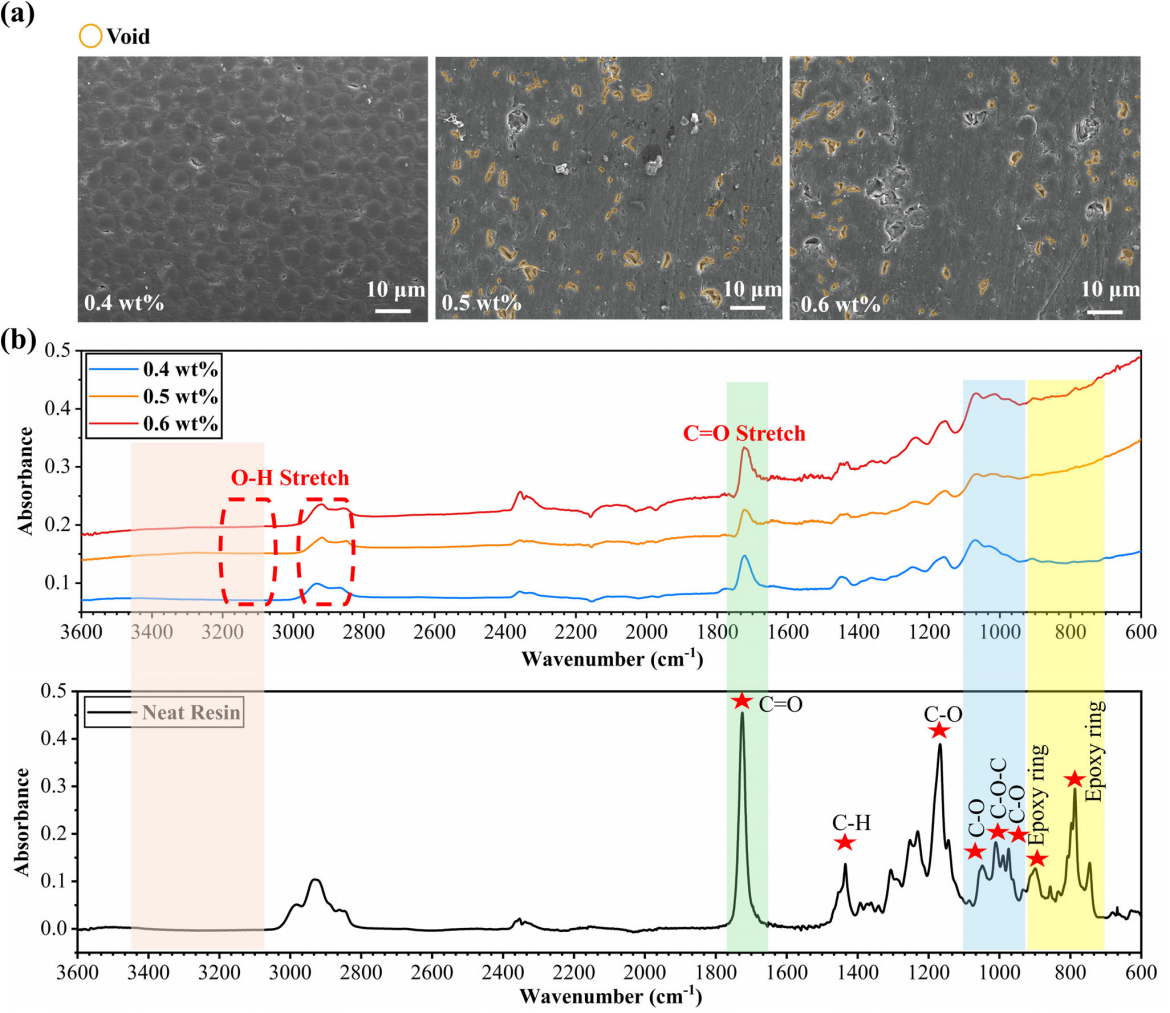
Micro void formation and FTIR analysis of CFRP laminates fabricated via EPFP method with varying photoinitiator (PI) content. a) SEM images of CFRP laminates with varying PI content. The 0.4 wt% PI laminate shows significantly lower porosity compared to 0.5 and 0.6 wt%. b) FTIR spectra of the frontal resin laminates with varying PI content. The spectra related to 0.5 and 0.6 wt% laminates highlight the presence of side reactions compared to 0.4 wt% PI samples.

With an aim to optimize the EPFP process, we studied the effect of photoinitiator (PI) content (0.4, 0.5, and 0.6 wt%) on FP characteristics, void contents, and thermomechanical performances of flat beam CFRP laminates, given in **Figures** **3** and **4**. The detail temperature profiles for different PI wt% are illustrated in Figures S4–S6 (Supporting Information). Noted that the thermocouples were strategically placed to monitor the temperature profiles within the laminate (see Figure 3a,c): two were positioned at the top regions of the fabric stack, one at the left and the other in the center at 2.1 mm vertical distance from the top surface, while two others were located at the bottom, with one in the center and the other on the right at 4.1 mm vertical distance from the top surface. The results presented in Figure 3b indicate that front temperature and front velocity in both through‐thickness and in‐plane directions showed clear upward trend as the PI content increased from 0.4 to 0.6 wt%. At 0.4 wt% PI, front temperature stabilized at 192.72 °C with a through‐thickness velocity of 1.19 mm s^−1^. A more steady and controlled move of the front at this concentration suggests a balance of the reaction kinetics so that heat‐generation and heat‐dissipation take place evenly, which minimizes thermal gradients. With the addition of PI from 0.4 to 0.5 wt%, this increased the system reactivity, and the front temperature rose to 197.95 °C, with the through‐thickness velocity increasing to 1.30 mm s^−1^. The increase in the front speed is due to the availability of increased exothermic heat generated during the FP. It attained a maximum temperature of 201 °C and the increased velocity through thickness up to 1.54 mm s^−1^ as against that of the 0.4 wt% PI, indicating further increase as compared to 0.4 wt% PI. The in‐plane velocity also experienced obvious increases. The in‐plane velocity was recorded as 6.02 mm s^−1^ at 0.4 wt% PI. The measured velocity raised up to 7.33 mm s^−1^ at 0.5 wt% PI before further attaining 8.26 mm s^−1^ at 0.6 wt% PI. These observations reflect the attainment of faster heat generation and reaction progress through the high PI content that propels the front in all directions. However, the data measured for front temperature and in‐plane velocity exhibited further variations with the higher PI levels recorded. The greater reaction rates exhibited for 0.5 and 0.6 wt% PI cause localized overheating, which is indeed destabilizing in the propagation of the front.

To explain how PI content influences the propagation process, delay times taken for the front propagating through the laminate thickness are presented in Figure 3c. The delay time was given in relative to the mid‐top position (*y =* 2.1 mm), referring to the duration the front requires to travel from the mid‐top position to three other positions. For 0.4 wt% PI, the delay time remained relatively uniform through the laminate with −4.60 s for the left‐top position (*y =* 2.1 mm) and 3.60 s for the right‐bottom position (*y =* 4.1 mm). The uniformity is attributed to consistent delay times, averaging ±4.00 s between the left‐top and right‐bottom positions. In other words, the front required a similar duration to travel from left to right (or vice versa) at both the top and bottom sides, indicating uniform heat dissipation and a stable reaction front. Increasing the PI content to 0.5 wt% resulted in a similar propagation pattern, but with a noticeable reduction in delay times. The delay time started at −5.05 s at the left‐top position (*y =* 2.1 mm) and reduced to 2.25 s at the right‐bottom position (*y =* 4.1 mm). The shorter delay times in both the in‐plane (horizontal direction from top‐left to right‐bottom) (7.30 s) and through‐thickness (1.35 s) directions compared to the 0.4 wt% PI laminate (8.20 and 1.60 s, respectively) reflect increased front velocity and higher reaction kinetics due to the higher availability of PI. However, this increased speed of propagation was accompanied by sharper thermal gradients, thus leading to a deteriorated front stability. At 0.6 wt% PI, delay time further decreased compared to 0.4 and 0.5 wt%, respectively, beginning at −4.60 s at the top (*y =* 2.1 mm) and reaching at 2.15 s at the bottom position (*y =* 4.1 mm). The total delay time difference across the in‐plane direction was 6.75 s, and through the thickness, it was 1.15 s. The faster front propagation across the laminate thickness at 0.5 and 0.6 wt% PI reflects the difficulties in maintaining front stability under fast curing conditions due to the generation of localized heat in a shorter time, which disrupts the balance between heat generation and heat loss during the FP process, ultimately compromising the steady propagation of the polymerization front.^[^ ^26^, ^27^ ^]^

To provide information on the relationship between reaction kinetics and quality of microstructure, the degree of cure and porosity is taken into account for all three levels of PI and is presented in Figure 3d. Note that degree of cure and porosity were evaluated from three different cross‐section cuts taken at various positions along the length of the laminate. A detailed evaluation of degree of cure and porosity are provided in Figures S7 and S8 (Supporting Information). For the 0.4 wt% PI, porosity was taken to be 0.80%, while the degree of cure was found to be 89% (±8%) which indicated a balance in the curing process. The slower front propagation velocity at this PI concentration gives enough time for heat to diffuse uniformly, thus allowing polymerization to be complete while avoiding defects and releasing side reactions. At 0.5 and 0.6 wt% PI, the degrees of cure were noted to be at 50% and 63%, respectively, with wide deviations of 38% and 34%, respectively, due to exothermic heat during the FP process.^[^ ^26^, ^27^, ^28^, ^29^ ^]^ This also agrees with the observed increase in the porosity values which had risen to 9.20% (0.5 wt% PI) and 7.80% (0.6 wt% PI), respectively, as depicted in detail in Figure S7 (Supporting Information). Noted that to minimize void formation during wet hand lay‐up, several measures were implemented. First, the resin was preheated to 50 °C to reduce its viscosity from 0.27 to 0.07 Pa s for improved fiber wetting, both resin and fabric were degassed to remove entrapped air, and a consolidation pressure of 8 MPa was applied throughout the EPFP process. These measures contributed to the low porosity (0.80%) observed in the 0.4 wt% PI samples, which were prepared using the same hand lay‐up technique as all other samples. The notable increase in void fraction (up to 9.20%) observed at higher PI concentrations is attributed primarily to intensified exothermic reactions and associated side reactions that cause volatile and gas entrapment, rather than to the lay‐up method itself. This emphasizes that the observed variation in porosity correlates strongly with increasing PI concentration, not with inconsistencies in the infusion or lay‐up process. Moreover, the measured laminate thickness for these samples ranged from 1.55 to 1.64 mm, with a mean of 1.60 mm and a standard deviation of 0.048 mm. This low variability indicates consistent consolidation and supports that flexural modulus differences stem from void content, rather than significant fiber volume fraction deviations.

In Figure 3e, the effect of the PI content is evaluated to comprehend how FP characteristics, such as porosity and degree of cure, affected the thermomechanical properties. Compared to laminates using off‐the‐shelf WS epoxy resin, flexural strength and modulus exhibited a significant advantage for laminates fabricated with EPFP, particularly at 0.4 wt% PI. Noted that, for completeness, the Supporting Information includes *T*_g_ and flexural data for long‐cycle thermally cured ECC laminates, which exhibit a flexural strength of 610.35 MPa and a flexural modulus of 70.19 GPa, values comparable to the neat frontal CFRP laminates. The flexural strength peaked at 683 MPa and modulus at 74.1 GPa at 0.4 wt% PI, constituting a very substantial improvement over WS epoxy laminate 537.40 MPa for flexural strength and 57.40 GPa for flexural modulus. This enhancement arises from the ability of the EPFP method to reach a remarkably high cross‐linking density (CLD) while restraining void formation, thanks to a balance between heat generation and heat dissipation. The increasing PI content in EPFP affected flexural strength and modulus negatively. The flexural strength fell as low as 503 MPa, with a modulus of 68.2 GPa at 0.5 wt% PI, and at 0.6 wt%, it marginally recovered to 579 MPa and 68.9 GPa, respectively. These trends reflect the trade‐off between faster polymerization and the formation of porosity due to excessive heat generation. While the EPFP samples at 0.5 and 0.6 wt% PI still outperform the WS epoxy laminate, the reduced flexural performance highlights the importance of controlling reaction kinetics to avoid generation of volatiles and side reaction. The large deviations in degree of cure at 0.5 and 0.6 wt% PI is attributed to the generation of trapped volatiles and side reactions, as confirmed by SEM images and FTIR spectra in Figure 4. The SEM images (Figure 4a) revealed a dramatic increase in micro void formation as the PI level increased from 0.4 to 0.6 wt% PI. With 0.4 wt% PI, the generation of exothermic heat and heat dissipation counterbalance and lead to a uniformly propagating front with little number of trapped volatiles and side reactions. Conversely, at the concentrations of 0.5 and 0.6 wt% PI, the fast rate of exothermic heat and higher generated heat promote side reactions occurring locally, such that they become trapped like gas bubbles. The trapped gases increase the porosity and make it difficult to accurately obtain the degree of cure across the length of the laminate.^[^ ^5^ ^]^

FTIR analyses (Figure 4b) bring additional insight for chemical changes happening before and after polymerization. The spectrum at higher PI contents (0.5 and 0.6 wt%) exhibit broadening and diminishing of the C═O stretching peak at 1725 cm⁻¹, accompanied by increased intensity in the O─H stretching region (3200–3600 cm⁻¹). While visual interpretation of the broad O─H stretching region (3200–3600 cm⁻¹) can be challenging, peak area integration across this region reveals a quantitative increase in O─H absorbance with increasing PI content (integrated areas: 0.8644 for 0.4 wt% PI, 3.2847 for 0.5 wt% PI, and 3.4638 for 0.6 wt% PI). This trend suggests an increased formation of hydroxyl species at higher PI concentrations, potentially due to enhanced radical‐induced side reactions or changes in hydrogen bonding within the polymer network. Careful examination of the C═O stretching peak at ≈1725 cm^−1^ also reveals partial differences. The 0.4 wt% PI sample displays a relatively well‐defined, symmetric C═O peak. In contrast, both the 0.5 and 0.6 wt% PI samples exhibit clear asymmetry in this peak. This distortion in peak shape indicates changes in the local chemical environment of the carbonyl groups. We attribute this loss of symmetry and any apparent diminishing of the peak primarily to structural modifications induced by increased free radical activity at higher PI concentrations. This indicates the presence of hydroxyl groups formed through epoxy ring‐opening and radical side reactions. The excess thermal energy generated at higher PI concentrations accelerates the reaction rate, leading to chain scission, free radical reactions, and branching. Radicals attack C═O and C─H bonds, resulting in the formation of additional hydroxyl and aliphatic groups, which disrupt the polymer network. More specifically, the excessive exothermic heat is induced to every monomer. With too many monomers started to be polymerized into prepolymers, the chances that the end‐group cations which are terminated by an anion to a final polymer chain will be higher, hence resulting in short polymer chain length and reduced mechanical properties (see Figure 3e).^[^ ^26^, ^27^, ^29^, ^30^ ^]^

The trend in flexural property is similar to the CLD, which reflects the polymer network creation and indicates the superiority of EPFP over traditional curing methods. The detailed DMA tensile results for neat frontal resin are given in Figures S9–S12 and Table S2 (Supporting Information). The CLD is calculated utilizing the DMA tensile results. The storage modulus was consistently taken at 160 °C for the calculation of CLD. This temperature was chosen specifically for this study as an alternative to the common *T*_g_+30 °C temperature, since not all samples reached the stable plateau in the storage modulus, which is usually required in the literature.^[^ ^31^ ^]^ The CLD was maximized with increasing PI up to 0.4 wt% value of 2756 mol m^−3^, indicating well‐structured, tightly cross‐linked polymer obtained by the progressive polymerization. This value was much higher than that of the off‐the‐shelf WS epoxy laminate with a lower CLD of 2023 mol m^−^^3^, verifying that EPFP was much more effective in creating a stronger network structure over a much shorter processing time. With an increase in PI content to 0.5 wt%, the CLD sharply decreased to 1593 mol m^−^^3^, despite a higher reaction rate compared to 0.4 wt% PI. The lower yield is due to the formation of side reactions and short polymer chains as a result of the large amount of exothermic heat during the FP process (Figure 4). These effects led to the disruption of the polymer network and resulted in inferior flexural properties. The CLD (1833 mol m^−^^3^) recovered measurably at 0.6 wt% PI (compared to 0.5 wt%), indicating a better degree of cure. However, because of the higher porosity associated with rapid front propagation at this concentration, the advantages gained from more effective cross‐linking were not sufficient to yield improved overall performance. In contrast, the CLD of WS epoxy laminate suggests that while prolonged thermal curing achieves curing completeness, it is less efficient in developing a dense polymer network compared to EPFP, emphasizing the advantages of the controlled front propagation observed at 0.4 wt% PI.

The highest glass transition temperature (*T*_g_) in Figure 3e indicated further improvement in the curing efficiency for EPFP laminates (see Figures S13–S16, Supporting Information, for details). The *T*_g_ value exhibited a sharp rise to 215.2 °C at 0.4 wt% PI, in contrast to only 56 °C in WS epoxy specimens. The higher *T*_g_ bears testament to the efficient cross‐linking provided by EPFP that in fact increases the thermal stability of laminates. *T*_g_ values of 0.5 wt% PI (212.6 °C) and 0.6 wt% PI (210.7 °C) were found to be still quite raised in comparison to WS epoxy but slightly lower than for 0.4 wt% PI due to the microstructural defects formed because of rapid heat generation.

Rate of manufacturing is another point demonstrating EPFP's advantage over conventional thermal curing. This parameter is demonstrated by the number of fabricated CFRP laminates (with 90 mm × 20 mm × 1.6 mm dimensions) per kWh (parts per kWh). Regardless of the amount of PI, as shown in Figure 3e, EPFP attained a production rate of 25 parts kWh^−1^ as opposed to a mere 1.11 parts kWh^−1^ for WS epoxy, indicating a tremendous leap. The energy efficiency attributable to FP rests on exothermic heat release, which lessens the demand of external heat and reduces curing duration.

### 2.4 EPFP‐Driven Synthesis and Application of Hybrid Resin Laminates

The study of neat frontal laminates underlines how well the EPFP process is performing and scaling in the production of high‐performance composites, given the further extension of such an approach using hybrid resin systems defines such advanced applicability. The accelerated curing time of FP incorporated with the mechanical robustness of commercial epoxies highlight the potential of EPFP in solving the inevitable problems in bulk manufacturing of composites that hybrid resin laminates undertake. This section focuses on EPFP applications in producing hybrid resin laminates with commercial resins in a significantly shorter period of time but achieving respectable mechanical and structural properties.

**Figure 5:**
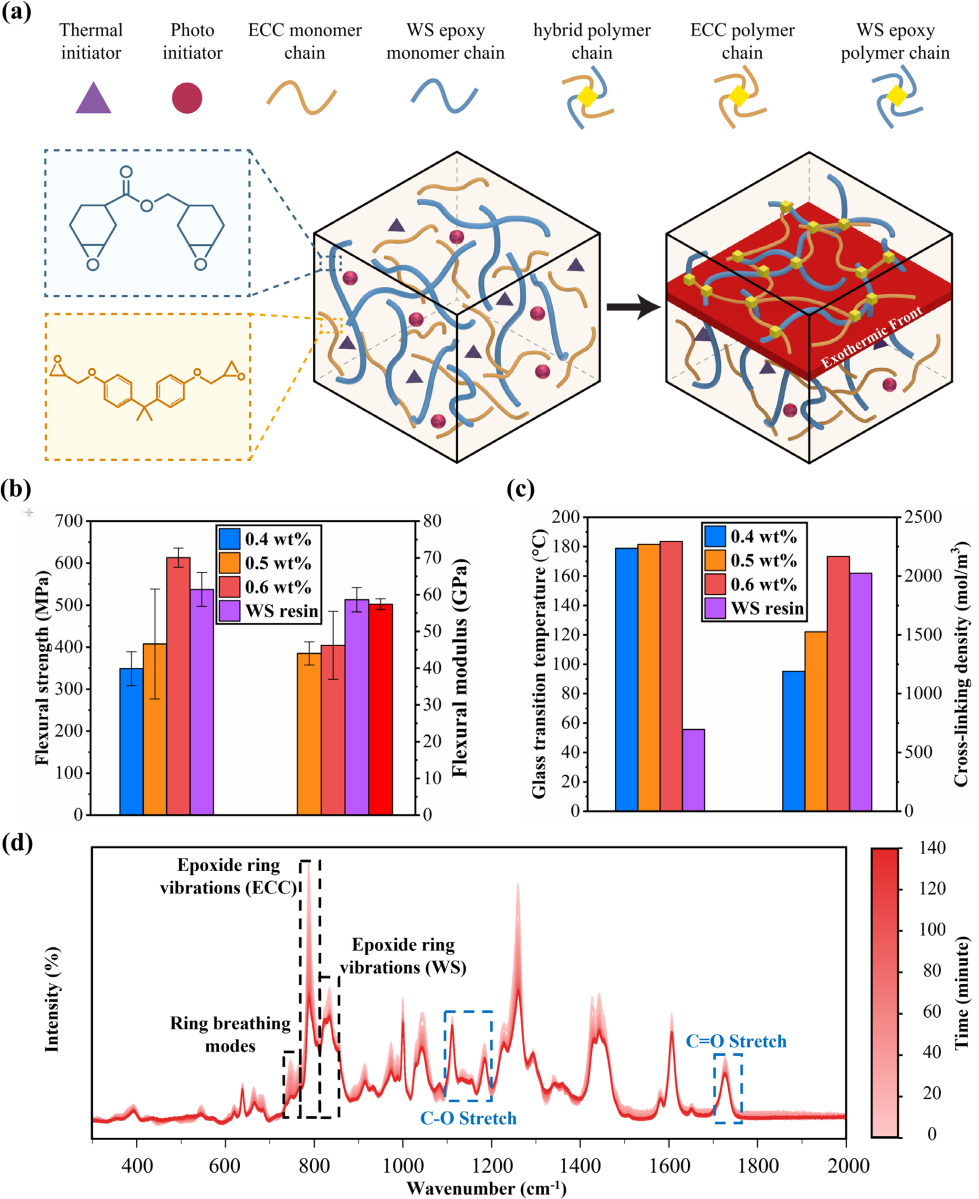
Thermomechanical properties and characterization of Exo‐press frontally polymerized hybrid‐resin CFRP laminates. a) Schematic of copolymer mechanism. The exothermic heat from frontal polymerization cross‐links the ECC frontal resin with the commercial off‐the‐shelf West System (WS) epoxy resin, generating a hybrid polymer network. b) Flexural strength and modulus of hybrid laminates at varying PI contents. The flexural strength and modulus highlight the improved performance at higher PI content. c) Glass transition temperature (*T*_g_) of hybrid resin‐based laminates under flexural DMA tests and cross‐linking density (CLD) calculated from the tensile DMA tests of neat hybrid resin samples. The increasing PI content improves the *T*_g_ and CLD compared to neat WS epoxy resin sample. d) Time‐lapse Raman spectroscopy of the 0.6 wt% PI hybrid resin. Real‐time evolution of frontal polymerization in the 0.6 wt% PI hybrid resin showed the ring‐opening reactions and network formation during the curing progression.

This application is illustrated by hybridizing the frontal resin with the off‐the‐shelf WS epoxy, which fosters a copolymer architecture. This architecture provides a substantial enhancement in thermomechanical properties of hybrid laminates over WS epoxy‐base laminate, as illustrated in **Figure** **5**. Figure 5a depicts how these dual‐network copolymers arise from the coexisting monomer matrices. As polymerization proceeds, these two networks entangle to form a robust copolymer network, thus boosting mechanical properties and thermal stability in comparison to WS epoxy‐base laminate. The data on flexural strength and flexural modulus in Figure 5b reveal the underlying role of PI levels (0.4–0.6 wt%) on those thermomechanical properties. The hybrid laminates achieve a flexural strength of 613 MPa and a modulus of 58.64 GPa at 0.6 wt% PI, performing better than samples with both 0.4 and 0.5 wt% and the WS epoxy‐based samples (537 MPa, 57.4 GPa). This improvement reflects a more extensive polymer network development in the presence of sufficient active radicals, creating additional cross‐links. On the contrary, in the case of 0.4 and 0.5 wt% PI laminates, there is a reduction in flexural strength compared to neat frontal resin‐based laminates. The 0.4 wt% PI had flexural strength of 348 MPa and modulus of 42 GPa, while the 0.5 wt% PI had flexural strength at 407 MPa and modulus 46 GPa, due to reduced exothermic heat availability and comparatively low cross‐linking density.

These flexural trends are further correlated to glass transition temperature (*T*_g_) and CLD in Figure 5c. The DMA tensile and flexural results related to neat hybrid resins and hybrid laminates, respectively, are provided in Figures S9–S16 and Table S3 (Supporting Information). We used the tensile DMA results for calculating the CLD of neat hybrid resin beams. As the PI content is sequentially raised from 0.4 to 0.5 wt% and 0.6 wt%, *T*_g_ also increases successively from 179 to 181 °C and from 181 to 184 °C, while CLD varies likewise from 1189 to 1529 mol m^−^^3^ and to 2167 mol m^−^^3^, respectively. The rise in both cases is in contrast to the values for the conventional WS epoxy‐based reference with *T*_g_ of 56 °C and CLD of 2023 mol m^−^^3^, which elucidates the better network formation with the hybrid resins. Furthermore, it is noteworthy that across these PI levels, the higher *T*_g_ of 184 °C at 0.6 wt% PI, compared to 0.4 and 0.5 wt% cases, represents both the densification of the network and the stabilization conferred by WS epoxy domains interspersed within the frontal resin matrix. Notably, the hybrid laminates in Figure 5 represent an advancement over the neat FP laminates from Figure 4 by addressing challenges at higher PI levels. Specifically, in neat FP systems, higher PI levels (e.g., 0.5 and 0.6 wt%) often generate excessive exothermic heat, leading to trapped gas, volatiles, and significant porosity (e.g., 9.2% at 0.5 wt% and 7.8% at 0.6 wt% in neat frontal resin‐based laminates). Thus, these steep temperature spikes in neat FP systems can entrap volatiles and create localized resin degradation and micro voids generation that can ultimately compromise the flexural properties and CLD. In contrast, hybridizing the frontal resin with WS epoxy resin effectively absorbs the excessive exothermic heat generated during the FP process through the WS epoxy, facilitating the formation of a robust copolymer matrix. This process mitigates the risk of side reactions and associated porosity, ensuring better structural integrity. So that at PI levels of 0.5 and 0.6 wt%, the neat FP laminates have uneven cross‐linking and substantial void formation, whereas the hybrid laminates have balanced polymerization process and superior network integrity at these higher PI levels. For instance, at 0.6 wt% PI, the hybrid laminate achieves a flexural strength of 613 MPa, which is higher than the neat FP laminate's 579 MPa at the same PI level, although its flexural modulus (58.6 GPa) remains lower than the neat FP's 68.9 GPa. At lower PI levels (0.4 and 0.5 wt%), both the flexural strength and modulus of the hybrid laminates are lower than those of the neat FP laminates. Thus, while the hybrid approach improves process stability and reduces defects, it does not consistently enhance flexural properties across all conditions. The co‐polymer network effectively leverages the stable epoxy domains from WS epoxy resin to enable fast curing with fewer defects, particularly at higher PI levels, even though the mechanical performance shows a trade‐off in certain cases. Further optimization of the hybrid system could involve exploring PI concentrations beyond 0.6 wt% to ascertain the upper limits of initiator effectiveness and its impact on the balance between processing stability and thermomechanical performance.

Raman spectroscopy was then utilized to further exhibit how hybridization governs frontal polymerization and enhances laminate quality. This technique provides critical insights into the chemical evolution of FP, capturing how distinct functional groups, including epoxide rings, aromatic phenyl bonds, ether linkages, and carbonyls, evolve under 0.4, 0.5, and 0.6 wt% PI (see Figures S17–S21, Supporting Information).^[^ ^32^ ^]^ Time‐lapse Raman spectroscopy of the hybrid resin at 0.5 wt% PI (Figure S18, Supporting Information) and 0.6 wt% PI (Figure 5d) offers a detailed view of how PI content governs the progression and completeness of frontal polymerization.

Raman spectra (Figures S19–S21, Supporting Information) confirm extensive ring‐opening in both ECC and WS epoxy, evidenced by the reduction of C─O─C epoxy ring vibration at 780 cm⁻¹ (ECC) and 810–950 cm⁻¹ (WS epoxy). Noted that WS epoxy alone remained unpolymerized under identical conditions, highlighting the importance role of ECC in initiating and propagating the front. The difference in reactivity arises because ECC cycloaliphatic epoxy is ring‐strained and influenced by carboxylate groups, whereas WS epoxy has a stabilizing aromatic backbone and less reactive functionality. When combined, ECC and WS epoxy form a complementary hybrid network. More specifically, highly reactive cycloaliphatic epoxy groups of ECC feature from rapid polymerization, producing a highly dense cross‐linked but brittle network. On the other hand, the aromatic backbone of WS epoxy along with epoxypropoxy groups yield a durable, chemically resistant matrix with more flexibility, counterbalancing ECC rigidity. The real‐time Raman spectroscopic analysis (Figure S18, Supporting Information; Figure 5d) on 0.5 and 0.6 wt% PI hybrid samples confirms progressive cross‐linking. Peak shifts in the 700–1400 cm⁻¹ region (C─O stretching, aromatic C─H bending) and the 1400–1800 cm⁻¹ region (carbonyl and aromatic C═C) reflect continuous network formation. Importantly, although the absolute intensity of the time‐lapse Raman signal decreases throughout the curing process (Figure 5d and Figure S18, Supporting Information) primarily due to polymerization‐induced changes such as increased opacity, densification, and reduced laser penetration depth, the relative intensity and shape of individual peaks within each scan provide clear evidence of evolving chemical structure (Figures S19 and S20, Supporting Information). At 0.6 wt% PI, near‐complete consumption of epoxides occurs, forming a dense polymer matrix, leading to improved flexural strength, modulus, and *T*_g_. In contrast, 0.5 wt% PI yields lower degree of cure which implied lower overall performance. Thus, the Raman data confirm the synergistic interplay between ECC and WS epoxy and the importance of tuning PI content for optimized frontal polymerization and robust laminate properties. A detailed discussion regarding the Raman analysis is provided in the Supporting Information.

### 2.5 Fabrication of Airfoil Skin via EPFP: Comparative Analysis of Cost, Energy, and Flexural Performance

**Figure 6:**
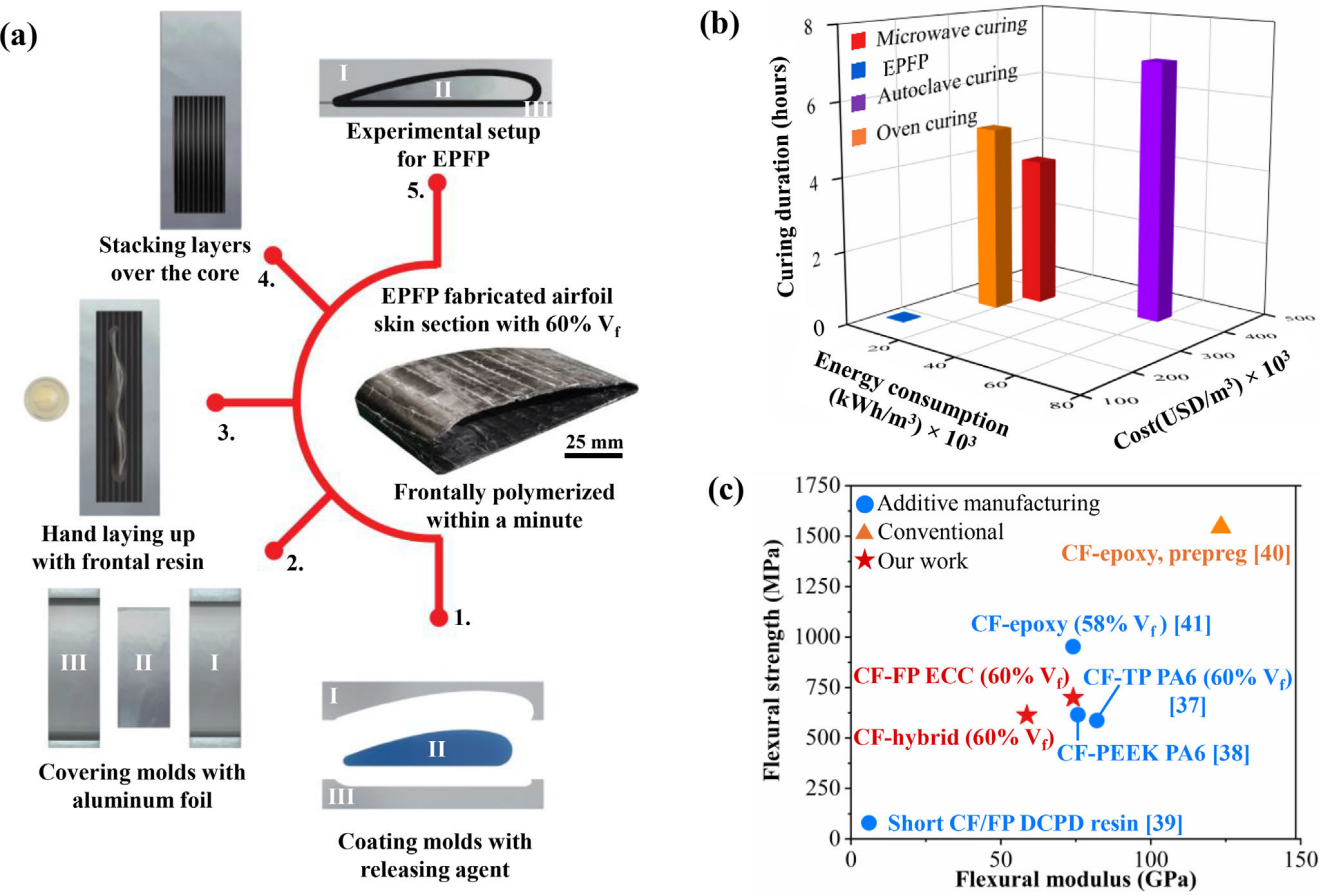
Demonstration of EPFP for an airfoil and benchmarking of cost–energy–mechanical performance of EPFP technique. a) Sequential steps in airfoil‐skin fabrication via Exo‐press frontal polymerization. The EPFP application yields a CFRP airfoil skin (60% *V*_f_) within a minute, depicting the capability of rapid and simple producing of complex geometry. b) Comparative assessment of energy consumption, cost, and curing duration. EPFP is evaluated compared to various composite manufacturing methods.^[^ ^33^, ^34^, ^35^, ^36^ ^]^ c) Flexural strength–modulus Ashby plot. The EPFP mechanical efficiency is benchmarked against different conventional and additive manufacturing methods.^[^ ^37^, ^38^, ^39^, ^40^, ^41^ ^]^

The ability to manufacture such complex geometries as airfoil components makes the EPFP approach one of the most attractive candidates for an efficient scalable and sustainable composite manufacturing technique. **Figure** **6**a shows a step‐by‐step EPFP process that can be employed for fabricating an airfoil skin and showcases its simplicity and versatility. Unidirectional carbon fiber sheets (50 mm × 200 mm) were stacked in 12 layers while applying hybrid frontal resin to obtain a *V*_f_ of 60%. These layers were stacked over the middle mold which the mold is covered with releasing agent‐coated aluminum foil. The assembled mold was then initiated by local thermal triggering in the hot press, leading to the sustainable self‐propagating EPFP process. The final airfoil skin component, shown in Figure 6a, demonstrates that the suggested process is capable of dealing with complicated geometries without needing sophisticated tooling or prolonged processing time.

EPFP's efficiency goes beyond geometrical flexibility, offering significantly greater cost and energy savings than traditional curing methods, as seen in Figure 6b.^[^ ^33^, ^34^, ^35^, ^36^ ^]^ Noted that all calculations corresponding to cost and energy consumption for all fabrication methods in Figure 6b are normalized to a unit of volume to ensure a consistent and fair comparison using the calculation methods in the literature.^[^ ^34^ ^]^ Used extensively in the aerospace industry, autoclave curing requires enormous energy and long cycle times to achieve homogenous curing. This results in high operating costs and restrains the scalability of this method. While not nearly as energy‐productive as autoclave curing, out‐of‐autoclave (OOA) process like microwave curing and thermal oven curing of prepregs still take several hours to process and include intricate temperature management systems. EPFP instead takes advantage of the exothermic nature of FP, removing the need for prolonged external heating and, therefore, greatly reducing its energy use and curing time. Specifically, in the EPFP process, while the same three‐part mold used in traditional prepreg/autoclave workflows is retained, the need for tooling is eliminated. Unlike autoclave curing, which requires vacuum bagging with bleeders, breathers, instrumented leak‐checked setups, and prolonged cure cycles, EPFP achieves consolidation and curing in a single step. The stack is placed in the mold, subjected to a brief thermal pulse, and frontally cures in one minute. This eliminates the use of vacuum bagging materials, feed‐throughs, and the autoclave itself, significantly simplifying the workflow. The reduction in tooling and handling complexity not only streamlines production but also minimizes the energy and time required. The process enables rapid manufacturing of large, complex components without requiring expensive autoclave systems or segmented fabrication. In addition, as shown in Figure 6b, EPFP's energy consumption per unit area is 94% lower than that of autoclave curing and 84% lower than OOA or thermal oven methods. In addition, EPFP's simplified infrastructure and reliance on localized heat triggering reduce costs by ≈44% compared to autoclave curing. The total curing duration for EPFP is one minute, compared to several hours for conventional methods, establishing EPFP as a highly time‐ and cost‐efficient alternative.

Beyond its economic and energy advantages, the flexural properties of EPFP laminates demonstrate a strong balance of performance and efficiency when compared to established methods, as shown in Figure 6c. To contextualize EPFP's performance with complex geometries (Figure 6a), additive manufacturing (AM) is included in the Ashby plot (Figure 6c), as both methods target rapid fabrication of intricate structures. Figure 6b, however, compares EPFP's curing efficiency specifically against traditional bulk thermal methods (autoclave, oven), making AM's broader process metrics less suitable for that chart. While EPFP utilizes press, it is distinct from conventional hot pressing that requires sustained external heat for its long cure. EPFP uses the press only for a brief thermal trigger (e.g., 15 s in our experiments), with the subsequent rapid (within a minute) cure driven by polymerization exothermic heat, significantly reducing energy and time compared to traditional thermal press curing. Laminates fabricated using EPFP with neat frontal resin (60% *V*_f_) achieved a flexural strength of 683.40 MPa and a flexural modulus of 74.1 GPa, outperforming continuous 3D printing of thermofiber (586.48 MPa, 82 GPa),^[^ ^37^ ^]^ laser‐assisted lamination of prepregs with 60% *V*_f_ (615.37 MPa, 75.65 GPa),^[^ ^38^ ^]^ and short carbon fiber composites (15% *V*_f_) with frontal polymerizable DCPD resin (80 MPa, 6 GPa).^[^ ^39^ ^]^ These results highlight EPFP's capability to produce high‐performance laminates with significant energy and cost savings. EPFP hybrid resin laminates (60% *V*_f_) achieved a flexural strength of 613 MPa and a flexural modulus of 58.6 GPa, reflecting a trade‐off between rapid curing and enhanced flexural strength. Despite this slight reduction in flexural modulus, EPFP hybrid laminates still outperformed traditional thermoset composites in simplicity and scalability. While advanced thermoset methods like APCM DA 409U delivered exceptional properties (1544.43 MPa, 123.42 GPa),^[^ ^40^ ^]^ their high energy demands and process complexity limit industrial feasibility. Similarly, thermoset tow‐prepreg extrusion of CFRP composites^[^ ^41^ ^]^ achieved 952.89 MPa in strength and 74.05 GPa in modulus but lacked the rapid curing, and scale‐up potential corresponding to EPFP method, due to demanding post compression and post curing of the 3D printed part and the slow fabrication process.

## 3 Conclusion

Exothermic‐press frontal polymerization is at the leading edge of composite manufacturing innovation. It redefined what is possible to be done in terms of manufacturing high‐performance CFRP laminates with unmatched efficiency, scale, and sustainability. By coupling the self‐sustaining nature of frontal polymerization with a tailored hybrid resin system, we successfully highlighted EPFP's rapid curing ability, which greatly decreases energy consumption and provides exceptional thermomechanical properties. The ability of this method to fabricate intricate geometries with high fiber volume fraction (*V*_f_ ≥ 60%), such as airfoil skins, demonstrates its flexibility for advanced industrial applications. This novel approach provides a cost‐effective alternative to traditional methods of manufacture because it eliminates the requirement for prolonged heating and complex process and tooling. Moreover, the capability to manufacture geometrically complex components such as airfoil along with hybrid resin signifies its application in aerospace, etc., pushing forward the application of EPFP into those sectors. The synergy between rapid polymerization and energy efficiency makes EPFP a very sustainable and scalable solution for next‐generation composite manufacturing, allowing it an open door for industrial‐scale adoption and innovation.

## 4 Experimental Section

### Materials

FP specimens used (3,4‐epoxycyclohexane)‐methyl‐3,4‐epoxycyclohexyl carboxylate (ECC; Sigma Aldrich, USA) as the epoxy monomer, a photo‐initiator (*p*‐(octyloxyphenyl)phenyl iodonium hexafluorostibate; IOC‐8 SbF6, AmBeed, USA), and a thermal initiator (benzopinacol; AAblocks, USA). Unidirectional carbon fabric (90 mm × 20 mm, 139 g m^−^^2^, 12K, FibreGlast, USA) was used as reinforcement. A hybrid resin system was prepared by mixing neat frontal resin with a commercial epoxy resin (WS epoxy 105, West System, USA) at a 1:0.3 mass ratio. Two reference laminates were also prepared: one with WS epoxy 105 and 206 Slow Hardener (West System, USA), and another using ECC cured with cycloaliphatic 4‐methylhexahydrophthalic anhydride (MHHPA, AAblocks, USA) and tertiary amine N,N‐dimethylbenzylamine (DMBA, AAblocks, USA)

### Fabrication Process

The EPFP process employed ECC containing 1 wt% benzopinacol (thermal initiator) and various PI concentrations of 0.4, 0.5, and 0.6 wt%. After a 10‐h dispersion in the dark, this frontal resin was mixed with WS epoxy 105 (1:0.3 ratio). The mixture and carbon fabric were degassed under vacuum (100 kPa) at 25 °C, with the resin preheated to 50 °C to reduce viscosity and the fabric dried at 150 °C. Twelve carbon plies (90 mm  ×  20 mm) were stacked in an aluminum mold 1.6 mm deep for a target fiber volume fraction of 60%. *V*_f_ is calculated using Equation (1)

(1) $$V_{f}=\frac{nA}{\rho h}$$

where *n* is the number of layers (12), *A* is the areal density of the carbon fiber sheet (139 g cm^−^^2^), *ρ* is the fiber density (1.745 g cm^−^^3^), and *h* is the thickness of the laminate (1.6 mm). The mold was set in a bench‐top press (Dabpress hydraulic heat press). Keeping the pressure at 8 MPa, the heat was applied for 15 s under the press followed by switching off the heat and allow the laminate to cure through the frontal polymerization process for 1 min, ensuring a fully cured laminate. Reference laminates based on WS epoxy 105 were cured at room temperature for 24 h under 8 MPa pressure, while those using ECC/MHHPA/DMBA were mixed (1.267:1 mass ratio) for 20 min, hot‐pressed, at 80 °C for 15 h.

### Computational Modeling

A 2D ABAQUS simulation coupled with home‐made subroutine^[^ ^3^, ^42^ ^]^ examined EPFP process in a 2.6 mm × 2.6 mm laminate cross‐section (60% *V*_f_). The heat conduction equation was modified to include exothermic polymerization, while cure kinetics followed an Arrhenius‐based autocatalytic model with parameters obtained from DSC. Triggering temperatures of 150–200 °C were applied to the top surface for 0.1 s to initiate the polymerization front. A fine mesh (18 µm^3^ DCAX4 elements) ensured accurate capture of temperature gradients, and insulated boundaries minimized heat loss.^[^ ^3^, ^42^ ^]^ The curing kinetics related to ECC frontal resin and material properties, including the carbon fiber and ECC resin, are shown in Tables S1 and S4 (Supporting Information).

### Differential Scanning Calorimetry

Differential scanning calorimetry (DSC, TA Q200) was used to determine curing kinetics. Prior to DSC, thermogravimetric analysis (TGA, TA TGA 550) determined the onset decomposition temperature (0.5% mass loss). Resin samples (3–5 mg) were sealed in T‐Zero pans, equilibrated at −20 °C, then ramped at 5 °C min^−1^ to 100–130 °C before a 50‐min isothermal hold. The Prout–Tompkins autocatalytic model yielded reaction parameters (*E*, *Z*, *m*, *n*) with residuals used to assess model fit.^[^ ^43^ ^]^ Full DSC plots are presented in Figure S1 (Supporting Information).

### Temperature Profiles

Front temperatures were recorded during EPFP in a 5‐mm‐thick laminate (36 plies, 60% *V*_f_). Four 0.1 mm K‐type thermocouples were embedded at depths of 2.10 and 4.10 mm, connected to a four‐channel thermometer (TC0520, Perfect Prime, USA) logging at 5 Hz. Measurements began upon hot pressing and continued through final cure. Figures S4–S6 (Supporting Information) detail the temperature evolution.

### Degree of Cure and Porosity Characterization

Fourier‐transform infrared spectroscopy (FTIR, Thermo Nicolet 6700) was used to quantify the epoxy conversion based on the oxirane‐to‐carbonyl peak ratio (789 vs 1724 cm⁻¹). SEM (JEOL JSM IT100 LV) cross‐sections of polished specimens (20 mm × 1.6 mm) revealed void content, measured with ImageJ by calculating the void‐to‐total area ratio (Figures S7 and S8, Supporting Information).

### Themomechanical Assessment

Flexural tests (ASTM D790‐03) were performed on EPFP and reference laminates at a 16:1 span‐to‐thickness ratio to evaluate flexural strength and modulus.^[^ ^44^ ^]^ Dynamic mechanical analysis (DMA, TA Q800) characterized glass transition temperatures (*T*_g_) and CLD (Figures S9–S16, Supporting Information). CFRP laminates (37 mm × 6.5 mm × 1.6 mm) were tested in three‐point bending mode with 0.1% strain from 15 to 350 °C at 3 °C min^−1^, while neat frontal and hybrid resins (15 mm × 6.5 mm × 1.6 mm) were tested in tensile mode at 1 Hz and 0.02% strain. *T*_g_ was taken from the tan(δ) peak, and CLD (ν_e_)^[^ ^31^ ^]^ was computed at 160 °C (*E*′ = 3ν_e_ *RT*).

### Raman Spectroscopy

Raman spectroscopy (Renishaw inVia with a 532 nm laser, 50× objective) was performed on hybrid resins (0.4–0.6 wt% PI) before, during, and after polymerization (Figures S17–S21, Supporting Information, and Figure 5d). Spectra were recorded in the 250–2000 cm⁻¹ range at ≈1.5 cm⁻¹ resolution. Real‐time scanning involved 10‐s acquisitions and acquisition interval of 35 s over a 3‐h window to capture ring‐opening and network formation. Measurements were conducted under dark conditions to prevent premature photo‐initiation, and data processing used Python.

## Conflict of Interest

The authors declare no conflict of interest.

## Author Contributions

A.T. carried out conceptualization, fabrication, characterizations, simulation, data analysis, writing the original draft, reviewing, and illustration. H.Z. contributed to porosity measurements. X.W. carried out the FTIR, Raman spectroscopy, and the related analysis. A.J.H. contributed to the preparation of figures and illustrations. K.D. performed DMA tests. K.F., Q.Q., and I.D.H. reviewed and edited the manuscript. Y.W. initiated, conceptualized, and directed the research. All authors discussed results and contributed to the final version of the manuscript.

## Supporting information



Supporting Information

Supplemental Video 1

## Data Availability

The data that support the findings of this study are available from the corresponding author upon reasonable request.
