# Supplementary material for: Efficient Exothermic Press toward Ultrafast and Scalable Manufacturing of Complex Polymer Composites
Source: Adv Sci (Weinh). 2025 Jul 17;12(38):e09336. doi: 10.1002/advs.202509336 (PMC12520577; doi:10.1002/advs.202509336)
Supplement: Supplementary file 1 — Supporting Information [file ADVS-12-e09336-s002.docx]

**Supporting Information**

**Efficient Exothermic Press Toward Ultrafast and Scalable Manufacturing of Complex Polymer Composites**

*Amirreza Tarafdar^1^, Haining Zhang^1^, Xinlu Wang^2^, Andrea J. Hoe^1^, Kaiyue Deng^3^, Kelvin Fu^3^, Quinn Qiao^1^, Ian D. Hosein^2^, Yeqing Wang^1*^*

^1^ Department of Mechanical & Aerospace Engineering, Syracuse University, Syracuse, NY 13244, USA

^2^ Department of Biomedical & Chemical Engineering, Syracuse University, Syracuse, NY 13244, USA

^3^ Department of Mechanical Engineering, University of Delaware, Newark, DE, 19716 USA

^*^ Corresponding author. Email: [ywang261@syr.edu](mailto:ywang261@syr.edu)

Raman spectroscopy analysis S1-S2

Curing kinetics model S3

Supplementary figures S4-S15

Supplementary tables S16

References S17

**1. Raman spectroscopy of hybrid resin**

Raman spectroscopy was utilized to further exhibit how hybridization governs frontal polymerization and enhances laminate quality. This technique provides critical insights into the chemical evolution of FP, capturing how distinct functional groups, including epoxide rings, aromatic phenyl bonds, ether linkages, and carbonyls^[1,2]^, evolve under 0.4 wt%, 0.5 wt% and 0.6 wt% PI (see Figures S17-S21). Time‐lapse Raman spectroscopy of the hybrid resin at 0.5 wt% PI (Figure S18) and 0.6 wt% PI (Figure 5d) offers a detailed view of how PI content governs the progression and completeness of frontal polymerization.

The Raman spectra before and after polymerization (Figures S19-S21) reveal significant reductions in peaks associated with key functional groups. For ECC, the C-O-C epoxy ring vibration at 780 cm⁻¹ decreases substantially, indicating ring-opening reactions essential for polymerization. Similarly, the epoxide group peaks of West system (WS) epoxy, observed in the 810–950 cm⁻¹ range, show a marked reduction, highlighting the successful consumption of reactive groups, a desirable outcome for cross-linking. Attempts to polymerize WS epoxy resin without ECC during EPFP failed, even under extended reaction times and intensified conditions, with WS epoxy remaining in a liquid state. This is further evidenced by the absence of the epoxide ring breathing peak at 610 cm⁻¹, a key indicator of epoxy ring-opening.

Importantly, although the absolute intensity of the time-lapse Raman signal decreases throughout the curing process (Figure 5d and S18) primarily due to polymerization-induced changes such as increased opacity, densification, and reduced laser penetration depth, the relative intensity and shape of individual peaks within each scan provide clear evidence of evolving chemical structure (Figures S19 and S20)

As illustrated in Figure 5a, both ECC and WS epoxy contain two oxirane rings, granting them bifunctionality and the ability to propagate polymer chains. However, their reactivity differs significantly due to structural factors. The cycloaliphatic epoxy rings in ECC are attached to a rigid six-carbon ring, introducing strain that increases the electrophilicity of the epoxide groups, making them highly reactive during cationic polymerization.^[3]^ Additionally, ECC carboxylate groups enhance reactivity through induction effects, drawing electron density away from the epoxide rings.^[4]^ In contrast, WS epoxy epoxide groups are attached to a flexible aliphatic chain and aromatic rings, which provide electronic stabilization, reducing their reactivity.^[4,5]^ The bulky aromatic groups in WS epoxy also create steric hindrance, further limiting access to its epoxide rings. This contrast underscores the complementary roles of ECC and WS epoxy in forming an effective hybrid resin system. The real-time Raman spectroscopic analysis (Figure S18 and Figure 5d) provides molecular-level insights into the polymerization reaction of 0.5 wt% PI and 0.6 wt% PI hybrid sample. Noted that, although the absolute intensity of the time-lapse Raman signal decreases throughout the curing process (Figure 5d and S18) primarily due to polymerization-induced changes such as increased opacity, densification, and reduced laser penetration depth, the relative intensity and shape of individual peaks within each scan provide clear evidence of evolving chemical structure (Figures S19 and S20). Significant changes were observed in the 700–1400 cm⁻¹ range, involving C-O stretching and C-H bending (aromatic) bonds. The C-O stretching peak at 1259.30 cm⁻¹ shifted to 1293.10 cm⁻¹ post-polymerization, indicating alterations in the bonding environment of oxygen-containing groups due to cross-linking. Similarly, C-H bending peaks (aromatic) in the 1150–1300 cm⁻¹ range showed intensity increases, such as the peak at 1182.73 cm⁻¹ rising from 0.41 to 0.76 in normalized intensity, reflecting stabilization of the aromatic framework within the polymer network. Furthermore, the disappearance and merging of peaks in the epoxy-related region (~780–950 cm⁻¹) confirms the consumption of epoxy groups during cross-linking, contributing to the formation of a dense, copolymer network. Additionally, the spectroscopic analysis in the 1400–1800 cm⁻¹ range reveals critical insights into the roles of carbonyl and aromatic groups in the polymerization process. The C=O stretching peak at 1726.73 cm⁻¹ shifted slightly to 1725.13 cm⁻¹ with a normalized intensity increase from 0.37 to 0.82, highlighting the involvement of carbonyl groups in network formation. The aromatic C=C stretching peak at 1605.83 cm⁻¹ showed a substantial intensity increase from 0.51 to 1.00, indicating enhanced conjugation and resonance stabilization due to the incorporation of aromatic monomers into a growing polymer network. Notably, the peak position remained unchanged, preserving the core aromatic structure.

The combination of ECC and WS epoxy creates a copolymer that effectively integrates the unique characteristics of both materials, resulting in exceptional thermomechanical characteristics compared to WS epoxy laminates. ECC, a multifunctional epoxy monomer with highly reactive cycloaliphatic epoxy groups, polymerizes rapidly through cationic mechanisms, forming a tightly cross-linked network. This dense network structure provides excellent dimensional stability and structural integrity, though it introduces brittleness. Conversely, WS epoxy, an epoxy resin derived from bisphenol-A diglycidyl ether, features an aromatic backbone and reactive epoxypropoxy groups.^[6,7]^ Its molecular structure enables the formation of a three-dimensional polymer matrix that is highly durable and chemically resistant, complementing ECC's rigidity with added flexibility and reduced brittleness. The rigid cycloaliphatic epoxy backbone of ECC contributes high cross-link density, ensuring the polymer stability under stress, while the ether (-C-O-C-) linkages and aromatic groups in WS epoxy enhance its resilience and facilitate a more adaptable network.

**2. Curing kinetics model**

The cure kinetics of the epoxy system were modeled using a modified autocatalytic rate equation that accounts for both the temperature dependence and the extent of cure:

$\frac{d\alpha}{dt}=A\exp\left( -\frac{E}{RT} \right) \alpha^{m} {(1-\alpha)}^{n} f(\alpha)$ (1)

where 𝛼 is the degree of conversion, 𝑇 is the absolute temperature (K), and 𝑅 is the universal gas constant. and 𝐴, 𝐸, 𝑚, and 𝑛 are kinetic parameters determined via nonlinear regression of isothermal DSC data (Figure S1). The exponential Arrhenius term captures the temperature dependence, while the $\alpha^{m} {(1-\alpha)}^{n}$ term accounts for the autocatalytic reaction mechanism typical in epoxy–amine systems.^[8]^ To incorporate the reduction in cure rate caused by vitrification and diffusion limitations at high conversion, a diffusion factor 𝑓(𝛼) was included:

$f\left( \alpha\right)= \frac{1}{1+exp (\alpha- \alpha_{C})/\Delta\alpha}$ (2)

Here, 𝛼_C_ is the critical conversion beyond which diffusion control becomes significant, and Δ𝛼 is a smoothing constant. This correction reflects the transition from chemically controlled to diffusion-controlled kinetics, as observed in dielectric and DSC studies of epoxy systems.^[9,10]^ Kinetic parameters were obtained by fitting isothermal DSC data using nonlinear regression.

All kinetic parameters were extracted by non-linear regression of the isothermal DSC data, and the resulting values (Table S1) closely match those reported by Fournier et al.^[9]^ and Xie et al.^[10]^, confirming the robustness of our fit.

**3. Supplementary figures**





**Figure S1.** DSC curve of the 0.4 wt% PI frontal resin tested at 100 °C to 120 °C, showing heat flow as a function of time during isothermal curing.

**Figure S2**. Evolution of the degree of cure at various time steps during the polymerization process under 150 °C triggering temperature.

**Figure S3**. Evolution of the degree of cure at various time steps during the polymerization process under 175 °C triggering temperature.





**Figure S4.** Evolution of front temperature for 0.4 wt% PI neat frontal laminate. Full temperature profiles from the onset of triggering, showing temperatures recorded at the top-right, top, bottom, and bottom-left points of the.





**Figure S5.** Evolution of front temperature for 0.5 wt% PI neat frontal laminate. Full temperature profiles from the onset of triggering, showing temperatures recorded at the top-right, top, bottom, and bottom-left points of the laminate.





**Figure S6.** Evolution of front temperature for 0.6 wt% PI neat frontal laminate. Full temperature profiles from the onset of triggering, showing temperatures recorded at the top-right, top, bottom, and bottom-left points of the laminate.

**Figure S7**. Porosity measurement across different sections of EPFP fabricated laminates. (a) Schematic of sample segmentation at x=25 mm, x=50 mm, and x=75 mm along the laminate length for porosity (b) Measured porosity for three specimens with 0.4 wt%, 0.5 wt%, and 0.6 wt% PI across the corresponding sections.

**Figure S8.** Degree of cure calculation across different sections of EPFP fabricated laminates for three specimens with 0.4 wt%, 0.5 wt%, and 0.6 wt% PI.

**Figure S9.** Dynamic Mechanical Analysis (DMA) tensile testing of neat resin beam sample with 0.4 wt% PI (frequency: 1 Hz, strain: 0.02 %). (a) DMA tensile results for frontal resin (b) DMA tensile results for hybrid resin.

**Figure S10.** Dynamic Mechanical Analysis (DMA) tensile testing of neat resin beam sample with 0.5 wt% PI (frequency: 1 Hz, strain: 0.02 %). (a) DMA tensile results for frontal resin (b) DMA tensile results for hybrid resin.

**Figure S11.** Dynamic Mechanical Analysis (DMA) tensile testing of neat resin beam sample with 0.6 wt% PI (frequency: 1 Hz, strain: 0.02 %). (a) DMA tensile results for frontal resin (b) DMA tensile results for hybrid resin.

**Figure S12.** Dynamic Mechanical Analysis (DMA) tensile testing of neat West System epoxy resin sample (frequency: 1 Hz, strain: 0.02 %).

**Figure S13.** Dynamic Mechanical Analysis (DMA) flexural testing of CFRP laminate with 0.4 wt% PI (multi-frequency strain). (a) DMA flexural results for frontal resin laminate (b) DMA flexural results for hybrid resin laminate.

**Figure S14.** Dynamic Mechanical Analysis (DMA) flexural testing of CFRP laminate with 0.5 wt% PI (multi-frequency strain). (a) DMA flexural results for frontal resin laminate (b) DMA flexural results for hybrid resin laminate.

**Figure S15.** Dynamic Mechanical Analysis (DMA) flexural testing of CFRP laminate with 0.6 wt% PI (multi-frequency strain). (a) DMA flexural results for frontal resin laminate (b) DMA flexural results for hybrid resin laminate.

**Figure S16.** Dynamic Mechanical Analysis (DMA) flexural testing of reference CFRP laminate (multi-frequency strain). (a) DMA flexural results for West System epoxy resin laminate (b) DMA flexural results for hot-pressed cured ECC-based laminate.

**Figure S17.** Raman spectrum of the neat West System (WS) epoxy resin highlighting key epoxy and aromatic peaks.

**Figure S18.** Time‐lapse Raman spectroscopy capturing the real‐time evolution of frontal polymerization in the 0.5 wt% PI hybrid resin.

**Figure S19.** Raman spectrum of neat West System (WS) epoxy resin, ECC resin before polymerization, and hybrid resin with 0.6 wt% PI before and after polymerization.

**Figure S20.** Raman spectra of neat West System (WS) epoxy resin, ECC resin, hybrid resins with 0.4 wt%, 0.5 wt%, and 0.6 wt% of PI before polymerization.

**Figure S21.** Raman spectra of neat West System (WS) epoxy resin, ECC resin, hybrid resins with 0.4 wt%, 0.5 wt%, and 0.6 wt% of PI after polymerization.

**4. Supplementary tables**

**Table S1.** Curing kinetics characteristics of ECC frontal resin.

| Pre-exponential  coefficient (*A*) | Activation energy (*E*) | Orders of reaction | Diffusion  parameters |
| --- | --- | --- | --- |
| 8.55e15 (s^-1^) | 101.23 (kJ/mol) | *n*=1.72 | *C*=0.02 |
|  |  | *m*=0.8 | *α_c_*=0.96 |

**Table S2.** DMA tensile results of neat FP resin samples.

| **Sample** | **Temperature (°C)** | **Storage Modulus (MPa)** | **Loss Modulus (MPa)** | **CLD** |
| --- | --- | --- | --- | --- |
| 0.4 wt% | 160.02 | 27.18 | 1.47 | 2756.06 |
| 0.5 wt% | 160.07 | 13.70 | 2.01 | 1593.09 |
| 0.6 wt% | 160.13 | 19.81 | 2.34 | 1833.43 |

**Table S3.** DMA tensile results of neat hybrid resin samples.

| **Sample** | **Temperature (°C)** | **Storage Modulus (MPa)** | **Loss Modulus (MPa)** | **CLD** |
| --- | --- | --- | --- | --- |
| 0.4 wt% | 160.10 | 12.85 | 0.72 | 1189.51 |
| 0.5 wt% | 160.09 | 16.49 | 2.13 | 1526.47 |
| 0.6 wt% | 160.13 | 23.41 | 4.31 | 2166.91 |
| WS epoxy | 160.01 | 18.37 | 0.67 | 2023.35 |

**Table S4.** Material properties of carbon fiber^[11,12]^ and ECC^[13]^ frontal resin.

| **Material** | **Density (*ρ*)** | **Thermal Conductivity (*k*)** | **Specific heat**  **(*C_p_*)** | **Total enthalpy of reaction (*H_r_*)** |
| --- | --- | --- | --- | --- |
| Carbon fiber | 1760 (kg/m^3^)^[11]^ | 10.45 (W/m·K)^[11]^ | 795 (J/kg·K)^[11]^ | N/A |
| ECC resin | 1170 (kg/m^3^)^[13]^ | 0.20 (W/m·K)^[13]^ | 1400 (J/kg·K)^[13]^ | 260 (J/g) |

**References**

[1] Kotula, A.P., Woodcock, J.W., Gilman, J.W., and Holmes, G.A., *Polymer* **2023**, *278*, *125967*;

[2] Edwards, H., *Handbook of vibrational spectroscopy* **2002**, *3*, *1838-1871*;

[3] Jin, F.-L., Li, X., and Park, S.-J., *J. Ind. Eng. Chem.* **2015**, *29*, *1-11*;

[4] Aziz, T., Haq, F., Farid, A., Cheng, L., Chuah, L.F., Bokhari, A., Mubashir, M., Tang, D.Y.Y., and Show, P.L., *Carbon Letters* **2024**, *34*, *477-494*;

[5] Pineda, A.F.E., Garcia, F.G., Simões, A.Z., and Silva, E.L.d., *International Journal of Adhesion and Adhesives* **2016**, *68*, *205-211*;

[6] Markwart, J.C., Battig, A., Velencoso, M.M., Pollok, D., Schartel, B., and Wurm, F.R., *Molecules* **2019**, *24*, *3901*;

[7] Chen, C., *Progress in Adhesion and Adhesives* **2024**, *8*, *251-282*;

[8] Bilyeu, B., Brostow, W., and Menard, K.P., *Journal of materials education* **2001**, *23*, *189-204*;

[9] Fournier, J., Williams, G., Duch, C., and Aldridge, G.A., *Macromolecules* **1996**, *29*, *7097-7107*;

[10] Xie, H., Liu, B., Sun, Q., Yuan, Z., Shen, J., and Cheng, R., *J. Appl. Polym. Sci.* **2005**, *96*, *329-335*;

[11] Tarafdar, A., Jia, C., Hu, W., Hosein, I.D., Fu, K., and Wang, Y., *Compos. Part B Eng.* **2023**, *266*, *111029*;

[12] Wang, Y., *J. Appl. Polym. Sci.* **2022**, *139*, *e52735*;

[13] Mousavi, S.R., Estaji, S., Kiaei, H., Mansourian-Tabaei, M., Nouranian, S., Jafari, S.H., Ruckdäschel, H., Arjmand, M., and Khonakdar, H.A., *Polym. Test.* **2022**, *112*, *107645*;
